# The Genome of *Lolium multiflorum* Reveals the Genetic Architecture of Paraquat Resistance

**DOI:** 10.1111/mec.17775

**Published:** 2025-04-26

**Authors:** Caio A. Brunharo, Aidan W. Short, Lucas K. Bobadilla, Matthew A. Streisfeld

**Affiliations:** ^1^ Department of Plant Science The Pennsylvania State University University Park Pennsylvania USA; ^2^ Institute of Ecology and Evolution, University of Oregon Eugene Oregon USA; ^3^ Department of Crop Sciences University of Illinois Urbana Illinois USA

**Keywords:** genetic divergence analysis, genome annotation, genome assembly, genome‐wide association studies, quantitative trait loci, RNA‐seq

## Abstract

Herbicide resistance in agricultural weeds has become one of the greatest challenges for sustainable crop production. The repeated evolution of herbicide resistance provides an excellent opportunity to study the genetic and physiological basis of the resistance phenotype and the evolutionary responses to human‐mediated selection pressures. *Lolium multiflorum* is a ubiquitous weed that has evolved herbicide resistance repeatedly around the world in various cropping systems. We assembled and annotated a chromosome‐scale genome for *L. multiflorum* and elucidated the genetic architecture of paraquat resistance by performing quantitative trait locus analysis, genome‐wide association studies, genetic divergence analysis and transcriptome analyses from paraquat‐resistant and ‐susceptible *L. multiflorum* plants. We identified two regions on chromosome 5 that were associated with paraquat resistance. These regions both showed evidence for positive selection among the resistant populations we sampled, but the effects of this selection on the genome differed, implying a complex evolutionary history. In addition, these regions contained candidate genes that encoded cellular transport functions, including a novel multidrug and toxin extrusion (MATE) protein and a cation transporter previously shown to interact with polyamines. Given that *L. multiflorum* is a weed and a cultivated crop species, the genomic resources generated will prove valuable to a wide spectrum of the plant science community. Our work contributes to a growing body of knowledge on the underlying evolutionary and ecological dynamics of rapid adaptation to strong anthropogenic selection pressure that could help initiate efforts to improve weed management practices in the long term for a more sustainable agriculture.

## 1 Introduction

Agricultural activities have tremendously modified the landscape and ecological dynamics of its original inhabitants (Tilman 1999). The need to increase food production over the past 50 years has led to a drastic simplification of agroecosystems (Pretty 2018). Weed species have colonised these highly disturbed areas and now compete with crops for resources. Chemical control with herbicides is the main strategy used in modern agriculture, and overreliance on these agents has resulted in the widespread evolution of herbicide resistance (Heap 2025). The evolution of herbicide resistance is one of the greatest challenges to sustainably produce food, fibres and fuel (Peterson et al. 2018).

Resistance mechanisms in weeds are classified into two categories: target‐site and non‐target‐site (Gaines et al. 2020). The genetics of target‐site resistance has been studied extensively and involves alterations to the herbicide's target enzyme. By contrast, the genetic basis of non‐target‐site resistance is believed to be more complex (Suzukawa et al. 2021). Although it has been shown that non‐target‐site resistance can be caused by increased herbicide metabolism (Brunharo et al. 2019) or sequestration of the herbicide to the vacuole (Brunharo and Hanson 2017), the genetic controls remain poorly understood. Indeed, basic questions about the genetic architecture of non‐target‐site resistance, including the number of loci affecting the trait, their distribution across the genome and whether they involve changes in gene expression, often remain unanswered.

However, some recent progress has been made in this area. For example, Kreiner et al. (2021) found that non‐target‐site resistance to glyphosate in *Amaranthus tuberculatus* was polygenic, with over 250 loci scattered throughout the genome, while Cai et al. (2023) found 15 loci associated with cycloxydim resistance in *Alopecurus myosuroides* . Conversely, Murphy et al. (2021) found two large‐effect loci associated with non‐target‐site resistance to tembotrione herbicide in *A. tuberculatus* , and Giacomini et al. (2020) found a single locus associated with 2,4‐D resistance in *A. tuberculatus* . Thus, even though there are very few examples, results from the literature imply that the genetic architecture of resistance traits seems to vary based on the type of herbicide resistance and the species.

In addition, there is limited information available on the sources of the alleles that allow for rapid adaptation in invasive plants. Adaptive alleles can arise via new mutations, gene flow between populations, or following selection on alleles that pre‐existed in the ancestor (i.e., standing variation). Studies on rapid adaptive evolution to biotic and abiotic stresses in agroecosystems also suggest that these dynamics are organism‐ and stress‐specific [Reviewed in (Hawkins et al. 2019)]. For instance, new mutations can confer fungicide resistance (Estep et al. 2015; Frenkel et al. 2015), while insecticide resistance in insects has been shown to evolve from new mutations or standing variation (Hartley et al. 2006; Hawkins et al. 2019; Lynd et al. 2010). Recent research suggests that resistance to herbicides also depends on the specific resistance mechanism involved and can vary among populations or species. Kreiner et al. (2019) found that glyphosate resistance in *A. palmeri* , a broadleaf plant species, occurred recently via increased copy number of the *EPSPS* gene. Conversely, a different population in their study had increased copy number prior to the onset of glyphosate applications, suggesting that selection occurred from standing genetic variation. Interestingly, they also found that glyphosate resistance had a polygenic architecture, with adaptive loci distributed throughout the genome. Thus, studies of the genetic architecture of herbicide resistance provide excellent opportunities to explore classic questions in the genetics of adaptation.

*Lolium multiflorum* Lam. is a diploid, winter annual species native to Europe, temperate Asia and Northern Africa, but human activities over the past 200 years have caused its spread to all continents (Humphreys et al. 2009). It is a troublesome weed in agriculture, where it can cause drastic reductions in yield if not controlled (Appleby et al. 1976). This species has the two‐locus *SZ* self‐incompatibility mating system (Fearon et al. 1983), contributing to extensive genetic diversity in populations. Management of *L. multiflorum* is primarily performed with herbicides, and the recurrent use of a few herbicide chemistries has resulted in the evolution of > 70 herbicide‐resistant populations across 14 countries (Heap 2025). Populations evolved resistance to important herbicide chemistries via inhibition of key target site enzymes, such as 5‐enolpyruvylshikimate 3‐phosphate synthase (EPSPS) (Brunharo and Hanson 2018; Karn and Jasieniuk 2017), acetyl CoA carboxylase (ACCase) (Brunharo and Tranel 2023), photosystem I inhibitors (PSI) (Brunharo and Hanson 2017) and acetolactate synthase inhibitors (ALS) (Xu et al. 2025). Resistance to multiple herbicides in *L. multiflorum* has recently been documented from agricultural fields in the western US (Brunharo and Tranel 2023; Brunharo and Streisfeld 2022; Bobadilla et al. 2021; Brunharo and Hanson 2018), with clear evidence of widespread gene flow among populations, as well as repeated, independent herbicide resistance evolution. Herbicide resistance in *L. multiflorum* provides an invaluable opportunity to fill many fundamental knowledge gaps related to the underlying genetic and ecological mechanisms of rapid convergent adaptation to similar environments across empirical systems.

Our previous work has identified *L. multiflorum* populations that evolved resistance to paraquat, a powerful PSI inhibitor (Brunharo and Hanson 2017). Paraquat is an herbicide discovered in 1955 (Brian et al. 1958) that has been used extensively around the world for non‐selective weed control. Although it is considered highly toxic, and it has been banned in many countries around the world, it continues to be one of the most widely used herbicides in the United States (EPA 2023). Paraquat is actively taken up by plasma membrane‐localised transporters, where it must reach the chloroplast to inhibit photosynthesis (Hart et al. 1992a). Once absorbed, it inhibits the PSI by functioning as a preferential electron acceptor, diverting electrons from ferredoxin to O_2_ and generating reactive oxygen species (ROS) (Hawkes 2014). In plants susceptible to paraquat, this fast reaction overwhelms the endogenous ROS quenching mechanisms, leading to membrane peroxidation and cell death within hours of treatment (Bromilow 2004).

Although much remains unknown about the genetic and physiological mechanisms that evolved in natural weed populations, we have recently gained some insight into paraquat resistance mechanisms by studying *Arabidopsis* mutants (reviewed by Nazish et al. 2022). Given that paraquat's target site is localised in the chloroplasts, mechanisms that prevent or reduce its movement can limit its action. Xi et al. (2012) identified a loss‐of‐function mutation in *pqt24‐1*, a gene that encodes a plasmalemma‐bound ATP binding cassette (ABC), exhibiting reduced cell influx of paraquat. A knockout of *mrv1* (encodes a polyamine transporter) reduced cellular paraquat uptake (Fujita et al. 2012). Restriction of paraquat trafficking from the Golgi apparatus to chloroplasts has been observed in *par1* mutant lines (Li et al. 2013), which contain a non‐synonymous mutation in the gene that encodes *AtLAT4*, another member of the polyamine transporter superfamily. Enhanced paraquat tolerance can also be conferred by enhanced vacuolar sequestration and cellular efflux. An amino acid substitution in *DETOXIFICATION EFFLUX CARRIER* (*DTX6*), a member of the multidrug and toxic compound extrusion (MATE) family, was suggested to increase affinity to paraquat (Lv et al. 2021b). It should be noted that the doses of paraquat used in *Arabidopsis* mutants can be six orders of magnitude lower than those that resistant weeds can withstand (0.1 μM in tolerant *Arabidopsis*, 0.13 M in paraquat‐resistant *L. multiflorum* ); (Brunharo and Hanson 2017, Fujita et al. 2012), potentially suggesting that different mechanisms could be at play in naturally evolved weed populations.

Genetic resources in *L. multiflorum* remain limited. A draft genome from a forage variety of *L. multiflorum* has been created but remains highly fragmented (Copetti et al. 2021). Therefore, a more contiguous, chromosome‐level assembly would be an essential resource for dissecting the genetic basis of important traits, such as herbicide resistance. In this context, the three primary objectives of this study are to (1) assemble and annotate the first, full chromosome‐level reference genome for *L. multiflorum* , (2) elucidate the genetic architecture and gene expression changes associated with paraquat resistance in *L. multiflorum* , and (3) identify the evolutionary signatures of paraquat selection pressure in the genome. To do so, we use genetic mapping, a genome‐wide association study (GWAS), transcriptome analyses and population genomic scans for signatures of recent selection between resistant and susceptible populations. Elucidating the genetic architecture responsible for herbicide resistance can provide insights into how organisms respond to strong selection pressure, and it can help initiate efforts to improve weed management practices in the long term.

## 2 Material and Methods

### 2.1 Source of Plant Material

A total of three paraquat‐susceptible and three paraquat‐resistant *L. multiflorum* populations were used in this study (Figure S1), with two resistant sites originating in agricultural fields in Oregon (population L60 and L31; Bobadilla et al. 2021) and one in California (PRHC; Brunharo and Hanson 2018), two susceptible locations from fields in Oregon (L46 and SLB; Bobadilla et al. 2021), and a known susceptible cultivated variety from Oregon (GULF; Brunharo and Streisfeld 2022).

### 2.2 *Lolium multiflorum* Genome Assembly and Annotation

A *L. multiflorum* individual was grown from seed in autoclaved sand from a previously characterised susceptible population (population GULF; Brunharo and Streisfeld 2022) and hydroponically irrigated with half‐strength Hoagland's solution. A tiller was clone‐propagated to a larger pot filled with potting soil and grown to maturity. Leaf tissue was collected from plants for high‐molecular weight DNA extraction (Wizard HMW DNA Extraction, Promega, Madison, WI, USA). We used flow cytometry to estimate genome size relative to *Conyza canadensis* and *Solanum lycopersicum* . Genomic DNA was sheared with a Megaruptor 3. Sheared DNA was converted to a sequencing library with the SMRTbell Express Template Prep kit 3.0, prior to sequencing on 11 SMRT cell 8M for generation of long‐read PacBio HiFi reads on a Sequel IIe platform with 30 h movie time. The combined yield was 244 Gb of raw HiFi data. RNA was extracted from root, leaf, pistil and anther tissue with the RNeasy Plant Mini Kit (Qiagen, Germantown, MD, USA), and individual libraries were generated with the Iso‐Seq SMRTbell prep kit 3.0, generating 23 Gb of HiFi data. To improve the assembly, we used proximity ligation (Hi‐C) to capture the 3D structure of chromosomes with the restriction enzymes DpnII, DdeI, HinFI and MseI and generated 138 Gb of Illumina paired‐end sequences.

We generated an initial haploid assembly using *Hifiasm* (v. 0.19.2) (Cheng et al. 2021). HiFi reads were assembled in the integrated mode along with the Hi‐C data. This initial assembly was further refined with the *purge_dups* pipeline. Scaffolding was performed with *YaHS* (Zhou et al. 2022) after processing of HiC reads, following the Arima genomics pipeline (https://github.com/ArimaGenomics/mapping_pipeline). Manual curation was performed with *Juicebox* (Durand et al. 2016). Finally, we conducted another round of scaffolding with *RagTag* (Alonge et al. 2022) using the *scaffold* module to order scaffolds onto the genome assembly of the closely related *L. perenne* (Frei et al. 2021).

Full‐length transcript sequences were individually processed following the *Iso‐Seq* and *tama* pipelines (Kuo et al. 2020). Briefly, sequencing primer removal and demultiplexing was performed with *lima*, poly(A) tail trimming and concatemer identification and removal with *refine*, and clustering of reads and polishing with *cluster*. Processed reads from each tissue were aligned to the *L. multiflorum* reference genome with *minimap2* (Li 2018) and individually collapsed with *tama_collapse*, followed by merging with *tama_merge*. Repetitive elements were identified with *EDTA* (v2.1.0; Ou et al. 2019). The coding sequences generated in the previous step were provided to *EDTA* to improve repetitive element detection. *RepeatModeler* (Flynn et al. 2020) was also used to identify any remaining transposable elements missed by EDTA.

Genome annotation was performed with *Maker* (Campbell et al. 2014). We included the merged transcripts generated from the *Iso‐Seq/tama* pipeline, as well as protein sequences from *Brachypodium distachyon* , *Hordeum vulgare* and *L. perenne* obtained from Ensembl Plants for annotation based on protein homology. Repeats were masked with RepeatMasker, including the species‐specific library created with *EDTA*. We employed Augustus (Stanke et al. 2006) and SNAP (Korf 2004) ab initio gene predictors to train and predict genes, in addition to transcript, protein and repeat alignments. Functional annotation was performed by querying the protein dataset generated against the Uniprot and interproscan databases (Jones et al. 2014).

### 2.3 Phylogenetic Analysis of *L. multiflorum* and Other Closely Related Species

We studied the phylogenetic relationships of *L. multiflorum* with other related taxa in the Poaceae family. We used *OrthoFinder* (v2.5.4; Emms and Kelly 2019) to identify single‐copy orthologs from *L. perenne* , *L. rigidum* , *B. distachyon* , *Triticum aestivum* , *H. vulgare* , *Setaria viridis* , *Echinochloa crus‐galli* and *Oryza sativa* , and we produced alignments with *MAFFT* (Katoh et al. 2002). A phylogenetic analysis was performed with *RAxML‐NG* (Kozlov et al. 2019) and plotted with *ggtree* (Xu et al. 2022) and treeio (Wang et al. 2019). We used the CoGe (https://genomevolution.org/coge/) platform to obtain the pairwise synonymous mutation rates (*K*_*s*_) between *L. multiflorum* and *L. perenne* , *H. vulgare* , *T. aestivum* and *B. distachyon* . Divergence time was calculated based on a mutation rate of 5.76174 × 10^−9^ (De La Torre et al. 2017).

### 2.4 Quantitative Trait Locus (QTL) Mapping of Paraquat Resistance

We used QTL mapping to identify the genomic locations contributing to paraquat resistance. We generated an outcrossed F_2_ population segregating for resistance by crossing a paraquat‐susceptible sample (GULF) to a paraquat‐resistant sample (L60). After pollination, we covered the seedheads with pollination bags to avoid external pollen contamination, given that *L. multiflorum* is a wind‐pollinated, obligate outcrossing species. Individuals from the F_1_ population resulting from this cross were again crossed, generating the F_2_ mapping population. To phenotype paraquat resistance, plants from all generations were treated with 1682 g a.i. ha^−1^ of paraquat, which is equivalent to 50% more active ingredient than a farmer typically uses (approximately 7 L ha^−1^ of Gramoxone 2.0 SL; Syngenta Crop Protection LLC, Greensboro, NC, USA). This discriminating rate was chosen based on our previous research (Brunharo and Hanson 2017). Individuals were scored as dead or alive 14 days after treatment. Prior to paraquat treatment, leaf tissue was sampled from individual plants for DNA extraction. We isolated genomic DNA from 47 susceptible and 48 resistant F_2_ individuals and created individually barcoded nextRAD libraries (Russello et al. 2015) and sequenced on a Novaseq 6000 (2 × 150 bp reads).

Paired‐end reads were trimmed with *trimmomatic* (Bolger et al. 2014). Reads were aligned to the reference *L. multiflorum* genome with *bwa*, removed PCR duplicates with *samblaster* (Faust and Hall 2014), and sorted with *samtools* (Li et al. 2009). Sequencing data from resistant and susceptible individuals were pooled separately, and we then used *freebayes* (Garrison and Marth 2012) to identify SNPs. We used the QTL‐seq method, as implemented in *QTLseqr* (Mansfeld and Grumet 2018), to identify QTL regions associated with paraquat resistance (Takagi et al. 2013). A positive ΔSNP‐index that exceeds the 95% confidence interval suggests that identified alleles are significantly associated with the resistance phenotype. Raw sequencing data were deposited to Sequence Read Archive under BioProject PRJNA1046158.

### 2.5 Resequencing Data Generation and Analysis

While QTL mapping can provide information on the loci involved in the evolution of resistance, the large linkage blocks present in an F_2_ population can make it difficult to narrow down the genomic location(s) involved in the trait of interest. To complement the QTL analysis and to further explore the genetic architecture of paraquat resistance, we performed a GWAS from 94 individuals collected from six populations (19 GULF, 17 L31, 16 L46, 15 L60, 16 PRHC and 11 SLB individuals). At the 3‐tiller growth stage, plants were sprayed with lethal doses of paraquat (1682 g a.i. ha^−1^) as described above. Survival data (either dead or alive) was recorded two weeks after treatment, and this phenotypic data was used for GWAS. Genomic DNA from 94 individuals was sequenced on a Novaseq 6000 in 2 × 150 bp mode to generate 10× coverage (BioProject PRJNA1046158).

Paired‐end sequences were initially processed with *HTStream* (https://github.com/s4hts/HTStream). Processed files were aligned to the *L. multiflorum* genome with the *minimap2* module for short‐read sequences, and PCR duplicates were removed with the *MarkDuplicates* tool of *gatk* (Poplin et al. 2018). Variant detection was performed with *freebayes* (Garrison and Marth 2012). We used *bcftools* (Danecek et al. 2021) to retain biallelic variants with depths between 10 and 250 in at least 75% of the samples. To obtain an overview of the structural genetic diversity among *L. multiflorum* populations, we identified small variants with *manta* (Chen et al. 2016). Genome‐wide association analysis was performed with *GAPIT* (Lipka et al. 2012), with the Enriched Compressed Mixed Linear Model (ECMLM) (Li et al. 2014) and correcting for population structure with PCA eigenvalues (Figure S2) and a kinship matrix. Upon identification of statistically significant markers, annotated genes within 2 Mb upstream and downstream of the marker with the lowest significance were identified with *bedtools* intersect (Quinlan and Hall 2010).

### 2.6 RNA‐Seq Data Generation and Analysis and Weighted Correlation Network Analysis

To assess the effects of differences in transcription level conferring paraquat resistance, we compared gene expression levels among individuals from two independently generated F_3_ populations segregating for paraquat resistance that originated from either population PRHC or population L60. The F_3_ populations were created by crossing GULF with either PRHC or L60, and individuals of the F_1_ filial generation were pair‐crossed, generating the F_2_ populations. Because the resistance phenotype is dominant, F_3_ lines segregating for resistant and susceptible plants were identified by crossing multiple pairs of resistant F_2_ plants. F_3_ seeds were germinated and sprayed with paraquat at the 2‐leaf stage. F_3_ families that consisted of both resistant and susceptible plants were used for RNA sequencing to test for differences in gene expression. Leaf tissue was collected from independent F_3_ plants, in four replications per time point: 0 (immediately before application), 3, 6, 12 and 24 h after treatment. Tissue was snap frozen in liquid nitrogen. At each time point, leaf tissue was collected from four resistant and four susceptible individuals from each of the F_3_ populations, for a total of 40 plants sampled per F_3_ cross (resistant or susceptible over five time points in four replications). Plants were scored 7 days after application as dead or alive, and chlorophyll fluorescence was measured with a portable fluorometer (OS1p+, Opti‐Sciences, Hudson, NH, USA) at each collection time. RNA was extracted from samples with a commercial kit (RNease Plant Mini Kit), and 3'‐Tag‐RNA‐seq sequencing libraries were generated with the QuantSeq FWD kit (Lexogen GmbH, Vienna, Austria). Sequencing was performed on an Illumina Novaseq 6000 in 2 × 150 bp (BioProject PRJNA1046158). Forward sequences were filtered using *BBDuk* (https://sourceforge.net/projects/bbmap/). Filtered reads were aligned to the reference genome with *STAR* in *quantMode* (Dobin et al. 2012). Differential gene expression was quantified with the R package *edgeR* (Robinson et al. 2009) and *limma* (Ritchie et al. 2015), where contrasts were made within each time point, with resistant individuals compared to susceptible as the control group.

To determine if differentially expressed genes between resistant and susceptible individuals tended to be co‐expressed with genes with similar functions, we performed a Weighted Co‐Expression Network Analysis (WGCNA) with the *WGCNA* package (Langfelder and Horvath 2008) using chlorophyll *a* fluorescence as the response variable. This analysis complements the RNA‐seq in that it allows us to understand the transcriptome‐wide relationship among genes involved in paraquat resistance, rather than looking at genes individually. To identify any outlier samples in our dataset, we employed hierarchical clustering and subsequently used the *cutreeStatic* function of the *WGCNA* R package to eliminate the outliers (Langfelder et al. 2008). The appropriate soft‐threshold power was identified by performing the approximate scale‐free topology criterion using the *picksoftThreshold* function. We then derived a signed adjacency matrix through bi‐weight mid‐correlation and a signed topological overlap matrix through dissimilarity calculations. Genes were grouped into modules using hierarchical clustering, and we employed the dynamic tree‐cutting algorithm to partition genes into distinct modules. Next, we computed module eigengenes using the *moduleEigengenes* function, which allowed us to merge similar modules and pinpoint modules associated with paraquat resistance. To identify genes that exhibit a strong correlation with genes in modules linked to paraquat resistance (i.e., hub genes), we conducted an intra‐modular connectivity analysis. Hub genes were identified based on their module membership (ranging from 0 to 1, indicating overall connectivity) and their gene‐trait significance (determined by the Pearson correlation between expression and the trait).

### 2.7 Population Genomic Analyses of Selection on Resistance

To complement the genetic mapping approaches described above, we used population genomic information from the different susceptible and resistant populations to determine if there was evidence for recent positive selection among resistant plants. Under a model where the genetic architecture of resistance includes a few, large‐effect loci, the application of paraquat in agricultural fields is expected to result in signatures of a selective sweep surrounding the loci involved in resistance (Pritchard et al. 2010). These signatures of a hard selective sweep include reduced diversity and increased haplotype homozygosity (Maynard Smith and Haigh 1974). However, if resistance has a polygenic basis or if the genetic variation responsible for resistance existed in the population prior to the onset of paraquat application, functional variants would likely occur on multiple haplotypes, which would all increase in frequency in what has been described as a soft sweep (Hermisson and Pennings 2005; Kreiner et al. 2018; Barghi et al. 2020).

We began by calculating levels of genetic divergence (*F*_ST_) between the resistant and susceptible populations across the genome, with the expectation that locally elevated patterns of genetic divergence between susceptible and resistant populations would be associated with resistance. This is because positive selection results in a reduction in genetic diversity, which would lead to fewer heterozygotes in the population, elevating *F*_ST_ (Storz 2005). We estimated *F*_ST_ using the Weir method (Weir and Cockerham 1984) at each biallelic SNP after removing indels, as implemented in *VCFtools* (Danecek et al. 2021). These were plotted across the genome, and regions of exceptionally elevated *F*_ST_ were considered as potentially selected loci.

*F*_ST_ can be considered a relative measure of sequence divergence because its value is influenced by levels of genetic diversity within populations (Cruickshank and Hahn 2014). Although a region of locally elevated *F*_ST_ could be caused by reduced diversity in either (or both) susceptible or resistant groups, we expect that selection occurred in resistant individuals. Therefore, we expect to find locally reduced genetic diversity and fewer segregating sites (i.e., polymorphism) in resistant relative to susceptible individuals (Peter et al. 2012). In addition, we expect resistant individuals to show a higher average frequency of alternate alleles relative to the reference. The reference assembly is derived from a susceptible individual, so a higher frequency of alternate alleles in resistant individuals would be consistent with selection increasing the frequency of alleles responsible for resistance. Based on overlap between the genetic mapping and *F*_ST_ results, we focused these analyses on two regions on chr5 (see Section 3).

We also calculated the site‐specific extended haplotype homozygosity statistic in both regions. Due to a recent selective sweep, a rapid increase in the frequency of a beneficial mutation will result in elevated linkage disequilibrium, leading to extended patterns of homozygosity within haplotypes (Sabeti et al. 2002; Smith and Haigh 1974; Voight et al. 2006). However, in the absence of selection, we expect haplotypes to break down over time due to new mutations and recombination. We compared haplotypes from susceptible and resistant individuals by focusing on the two sites that had the highest *F*_ST_ in each of the two regions on chr5. We began by phasing the VCF file using Beagle (Browning and Browning 2007). We then extracted haplotype and marker information from each VCF file using the *data2haplohh* function of the *rehh* package (Gautier and Vitalis 2012). EHH was calculated from the marker and haplotype information for each of the population using the *calc_ehh* function in *rehh*.

To confirm the effects of selection and to begin to distinguish between hard and soft selective sweeps, we employed the H12 test (Garud et al. 2015). In this test, three statistics are calculated. H1 is the sum of the squares of the frequencies of each haplotype in a sample. H12 corresponds to the combined haplotype frequency of the first and second most abundant frequencies in a sample. H2 is the haplotype homozygosity after excluding the most abundant haplotype in a sample. Hard sweeps are expected to increase the frequency of a single haplotype, resulting in an elevated H1. By contrast, a soft sweep will result in multiple haplotypes increasing in frequency, leading to an elevated H2. Thus, a genome scan for elevated H12 is a powerful way to identify evidence of both hard and soft selective sweeps (Garud et al. 2015). We divided chromosome 5 into 100 SNP windows with a 50 bp step size and scanned for elevated H12 in each window. This test was performed separately for resistant and susceptible individuals, with the logic that selection for paraquat resistance should lead to an elevated H12 in resistant but not susceptible groups. Then, we focused on the 1% of windows with the highest H12 and examined the ratio of H2/H1. Under a soft sweep, we expect H2/H1 to be elevated, because multiple haplotypes will increase in frequency (Garud et al. 2015). Analyses were performed using scripts posted on this site: https://github.com/ngarud/SelectionHapStats/tree/master.

Finally, to evaluate if there was a parallel genetic architecture involved in resistance, we also asked if the three resistant populations we sampled were differentiated in the same regions of the genome. Elevated *F*_ST_ could be caused by only one or two resistant populations being differentiated from the susceptible populations, but this would not indicate a region that is always involved in resistance. To test this, we calculated *F*_ST_ in each 25 kb window across the genome between each pair of resistant and susceptible populations using the *popgenwindows.py* script (https://github.com/simonhmartin/genomics_general). For a window to be included in the analysis, we required a minimum of 25 SNPs to be present. For each of the nine pairwise comparisons, we counted the number of times each window was found in the top 1% of windows across the distribution of *F*_ST_ values, because the more often a window is found in the top 1% of *F*_ST_ values across comparisons, the more likely the same resistance haplotype is diverged from susceptible haplotypes. Codes for the population genomics analyses are available from GitHub at https://github.com/awshort/ryegrass/tree/main.

## 3 Results

### 3.1 A Chromosome‐Level Assembly and Annotation for *L. multiflorum*

**FIGURE 1:**
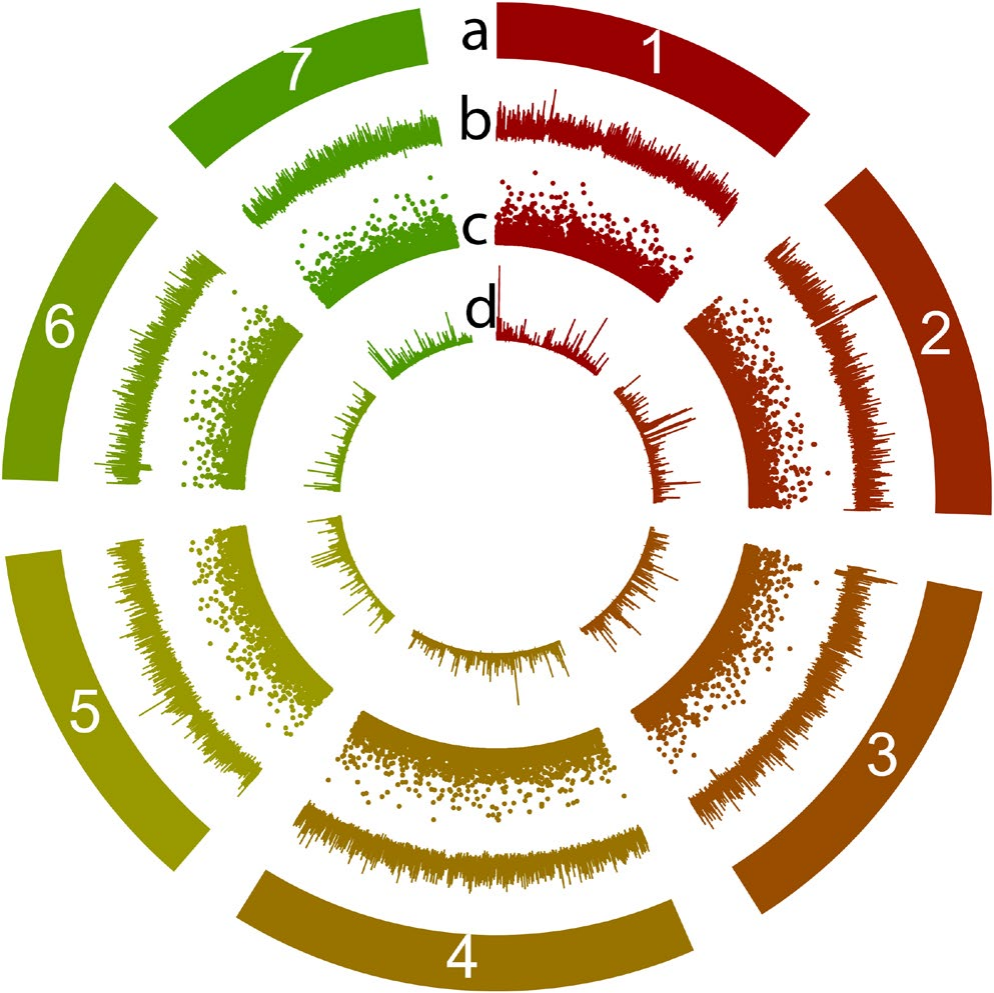
Circos plot of the *L. multiflorum* genome and its various features, plotted in 100 kb windows, where peak height represents feature frequency. Tracks represent (a) the seven chromosomes, (b) repetitive elements, (c) small variants (< 1000 bp), and (d) single‐nucleotide polymorphisms.

The first step towards elucidating the evolution of herbicide resistance in *L. multiflorum* was to assemble a high‐quality reference genome for this species (Figure 1). We generated 45× coverage long‐read data from DNA of an herbicide susceptible individual and used proximity ligation (Hi‐C) to generate a haploid assembly of the *L. multiflorum* genome (Table S1). The de novo assembly of the *L. multiflorum* genome resulted in 297 scaffolds, with *N*_50_ = 61 Mb and *L*_50_ = 12 (i.e., the length sum of 12 contigs contributes to at least 50% of the assembly). This initial assembly was improved by ordering scaffolds based on the synteny shared with the closely related species *L. perenne* , resulting in 7 chromosomes containing 2.55 Gb of nuclear content (90% of the genome; *N*_50_ = 363 Mb; Table S1). The genome size obtained *in silico* supports the flow cytometry estimates of 2.72 Gb haploid size. BUSCO analysis indicated that 93% of the single‐copy orthologs from the *poales_odb10* dataset were contained in the assembly. Approximately 83% of the DNA content is composed of repetitive elements (Table S2), as expected from a plant species with a large genome size (Schnable et al. 2009). We annotated the *L. multiflorum* genome with 23 Gb of Iso‐Seq data from leaf, root, pistil and anther tissue and identified 49,295 protein‐coding sequences.

In weed management, accurate species identification is crucial, because different species can exhibit distinct responses to efforts to control them. *L. multiflorum, L. perenne* and *L. rigidum* are often referred to, or treated as, a single species for basic biology and management (Scarabel et al. 2020; Yanniccari et al. 2020). The results of our single‐copy ortholog gene analysis from species closely related to *L. multiflorum* revealed their phylogenetic relationships. Although the chosen species are closely related, they are also clearly phylogenetically distinct, confirming morphological and life‐history studies performed elsewhere (Figure S3A) (Bararpour et al. 2017). In addition, based on the synonymous substitution rates (*K*_*s*_) between homologous gene pairs, we found that *L. multiflorum* appears to have diverged from *L. perenne* , *T. aestivum* , *H. vulgare* and *B. distachyon* approximately 5.4, 26, 27 and 29.4 M years ago (Figure S3B). These estimated divergence times largely agree with previous reports (Kumar et al. 2022).

### 3.2 Genetic Mapping Reveals the Genetic Architecture of Resistance

**FIGURE 2:**
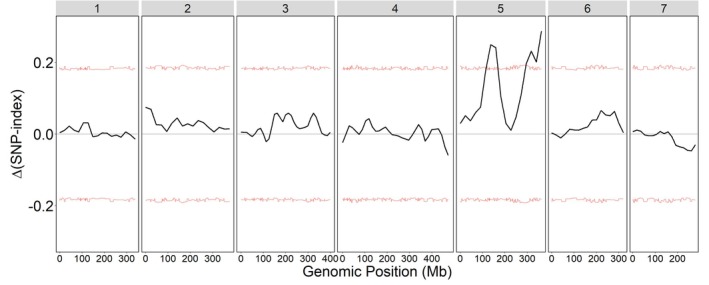
QTL‐seq analysis from an F_2_ mapping population between paraquat‐resistant and ‐susceptible *L. multiflorum* individuals. The numbers at the top represent the chromosome number. The red lines represent the 95% bootstrap confidence intervals based on 10,000 replicates used for significance testing.

A QTL analysis in an F_2_ mapping population identified regions of the genome segregating with the resistance trait (Figure 2). We identified two regions on chr5 that were significantly associated with paraquat resistance based on the ΔSNP‐index exceeding the 95% confidence interval. The intervals of the two detected QTL spanned positions 120,860,887–167,802,303, and 294,188,538–363,361,071 on chr5. This could suggest two separate loci on chr5 are responsible for paraquat resistance, but further analysis is necessary to determine the precise regions involved.

**FIGURE 3:**
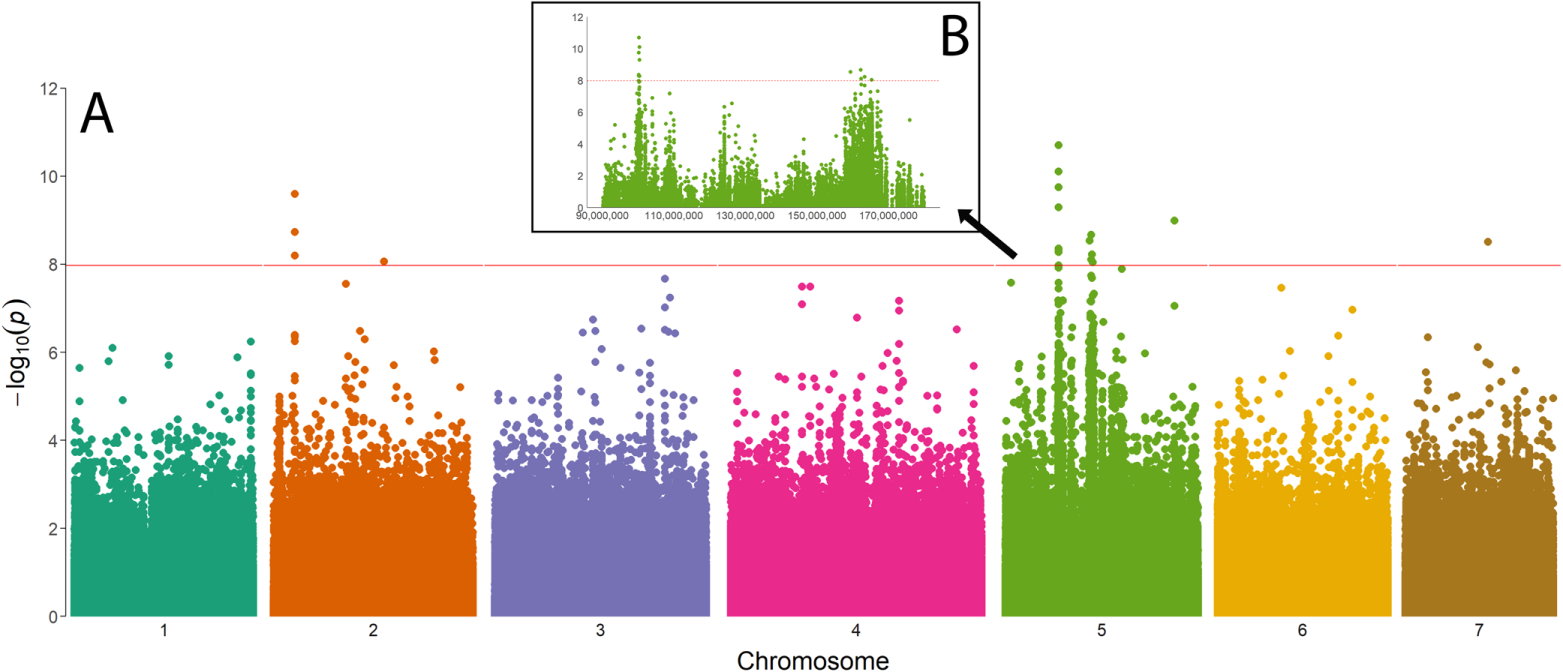
Genome‐wide association of paraquat‐resistance in *L. multiflorum* . (A) A total of 4,744,764 (*n*) makers were tested across the genome. (B) Close‐up of significant SNPs on chromosome 5. The red lines are Bonferroni threshold, calculated as 0.05/*n*.

To further explore the genetic architecture of resistance, we performed a GWAS by resequencing 94 *L. multiflorum* individuals to an average 10.8× coverage, and implemented statistical models in *GAPIT* (Lipka et al. 2012) to identify SNPs and INDELs associated with paraquat resistance. GWAS can take advantage of the genetic variation present across multiple individuals that have a longer history of recombination rather than the two parents used in the QTL mapping above. Moreover, determining the genomic regions where the QTL and GWAS analyses intersect would provide independent corroboration of potential candidates for resistance. The final GWAS dataset contained 3,385,096 SNPs and 1,359,667 INDELS, which resulted in an overall marker density of 1.7 variants per 1 kb. In total, we found 22 markers that were significantly associated with the difference between resistant and susceptible plants (Figure 3). Although five of these markers were statistically significant, they occurred as singletons, with no additional significant markers in those regions. Therefore, we focused only on the remaining 17 markers that localised to three distinct regions of the genome: one on chr2, and two on chr5. The three significant markers found on chr2 spanned a distance of only 289 bp (between positions 40,259,279 and 40,259,568). Of the remaining significant markers, eight of them localised to a 239 kb region on chr5 (between positions 100,183,991 and 100,423,860). The final five markers that were significantly associated with the phenotype also were located on chr5, between positions 159,409,415 and 165,297,925, a distance of 5.89 Mb. Although the first region on chr5 does not directly overlap with the QTL peaks (roughly 20 Mb away), this second region was contained within the first peak on chr5. Neither of these peaks on chr5 correspond to the second QTL peak at the end of chr5. Within each region, the marker with the lowest *p*‐value explained 5.9% (chr2), 20.1% (chr5, first region) and 35.3% (chr5, second region) of the phenotypic variation, but it remains possible that the variance explained by the two regions on chr5 is somewhat inflated due to linkage.

We further investigated these regions by extracting annotated genes in 2 Mb regions surrounding the marker in each region with the lowest P‐value (4 Mb total per region). The focal markers were position 40,259,568 of chr2, and 100,269,722 and 162,257,267 of chr5. A total of 214 genes were annotated near these three regions, of which 89 were on chr2, 64 near the first peak on chr5, and 61 near the second peak of chr5 (Table S3). There were several genes with known functions that could be involved in resistance to paraquat. For example, there were genes that had functions related to the response to oxidative stress, those that mediated intracellular transport, and genes known to respond to herbicides. Genes with potential roles in herbicide metabolism were identified, such as cytochrome P450s, as well as a polyamine oxidase gene that regulated polyamine intracellular concentration. Given that paraquat resistance is thought to be conferred by reduced herbicide movement, genes that encode membrane‐bound transporters are of particular interest. Specifically, we identified *SEC31B* (promotes the formation of transport vesicles from the endoplasmic reticulum) on chr2, *Transmembrane 9 superfamily member 1* (*TMN1*; involved in cell adhesion and phagocytosis in the secretory pathway), *Lysine histidine transporter‐like 8* (*AATL1*; a transmembrane amino acid transporter) and *VATP‐P1* (a V‐type proton ATPase) in the first region of chr5, and *NPF5.10* (*NRT1/PTR FAMILY*, a nitrate or di/tri‐peptide transporters), and *DTX10* (a member of the MATE family of proteins) in the second region of chr5.

### 3.3 RNA‐Seq Identifies Genes Differentially Expressed Between Susceptible and Resistant Plants

To complement the results from QTL‐seq and GWAS, we performed a differential gene expression experiment in two separate F_3_ populations segregating for paraquat resistance. Based on visualisation of a multidimensional scaling plot, there was a clear separation between mapping populations. Therefore, analyses were performed separately for each segregating population. A total of 55 genes were differentially expressed in the F_3_ population derived from PRHC, and 10 genes from pop60 (Table S4 and Figure S4). Of these, only two genes were found to be differentially expressed in both populations. One of these encoded a chloroplastic uncharacterized aarF domain‐containing protein kinase, and the other encoded a glutathione synthetase (GSH2). In both cases, the genes were expressed at higher levels in resistant individuals relative to susceptible ones, with *GSH2* being the most highly expressed gene in the dataset (Table S4). Interestingly, we found a strong over‐representation of differentially expressed genes across chr5. Of the 63 differentially expressed genes detected, 32 (51%) occurred on chr5. Among these, there was only one gene that was differentially expressed and found among the annotated genes near the GWAS hits (Figure S4). This gene encoded a protein with sequence similarity to the NPF5.10 protein from *Arabidopsis thaliana* and was expressed at a higher level in resistant plants from the PRHC‐derived population.

### 3.4 Gene Co‐Expression Networks Dissect the Coordinated Expression Patterns in Response to Paraquat

The network analysis grouped genes with similar expression patterns into separate modules, resulting in 18 modules identified in the population derived from L60 and 28 from PRHC (Figures S5 and S6, and Tables S5 and S6). The number of genes contained within the modules ranged from 74 to 3288 for L60, and from 38 to 3286 for PRHC. Similar patterns were observed across the modules that were positively correlated with chlorophyll *a* fluorescence (a proxy for paraquat resistance). Specifically, these modules contained co‐expressed genes with functions that included transmembrane transport, photosynthesis, glutathione biosynthesis, ABC transporters and superoxide metabolism (Figures S5 and S6). Hub genes included detoxification 21 (*DTX21*) and ABC transporters (*ABCB11*, *ABCA8*). In addition, *psbB Photosystem II CP47* and *atpI* ATP synthase subunit α were detected as hub genes in modules associated with photosystem responses and regulation (Figures S5 and S6, module ‘yellow’ and ‘tan’ in L60 and PRHC, respectively). These results further indicate that membrane‐bound transporters are responsive to paraquat application and could be involved in herbicide resistance or to minimise oxidative stress from paraquat application. Of the 60 major hub genes positively correlated with paraquat resistance, 19 (32%) were on chr5. Three annotated genes were found within the second QTL interval on chr 5: LOLMU_00024321 (Protein of unknown function), LOLMU_00024251 (Probable protein phosphatase 2C 20) and LOLMU_00027468 (Protein of unknown function).

### 3.5 Recent Positive Selection on Resistant Haplotypes

**FIGURE 4:**
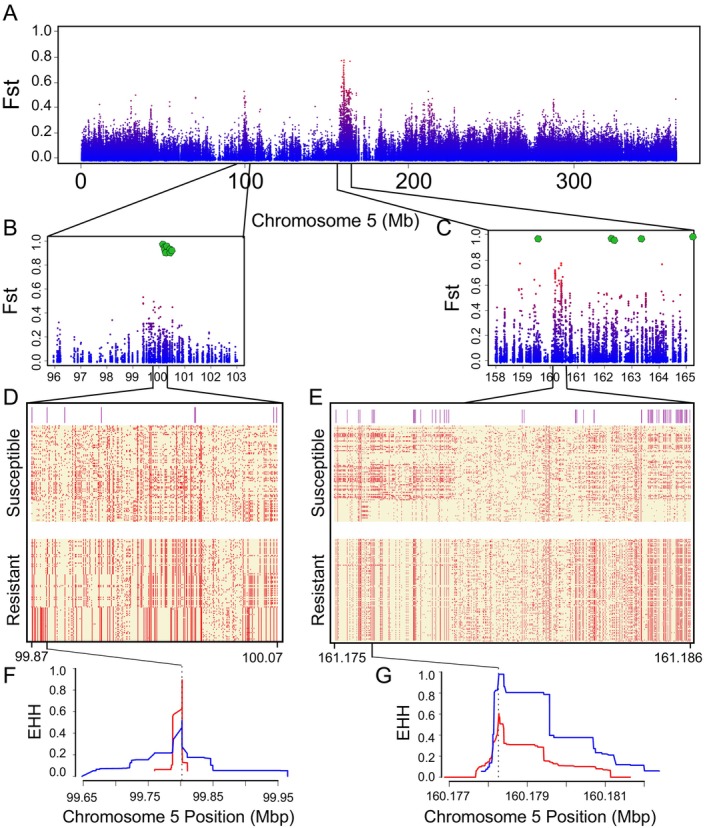
Recent positive selection on resistant haplotypes. (A) *F*_ST_ scan of biallelic SNPs across chromosome 5. The SNPs are colour‐coded based on their relative values across the entire genome (see Figure S7), with red indicating higher overall divergence. Panels (B) and (C) contain the same information as in panel (A), but they are zoomed in on regions 1 and 2. Green hexagons indicate the location of GWAS markers that were significantly associated with the resistance phenotype. (D, E) Each row is a haplotype from resistant or susceptible individuals, either for region 1 (D) or region 2 (E), and columns correspond to variant sites. Yellow boxes indicate the presence of the reference allele, while red boxes indicate the alternate allele. Purple lines above the haplotypes denote the sites where *F*_ST_ between resistant and susceptible populations is greater than 0.4. The range of sites included in each region is reported at the bottom of each panel, in Mb. (F, G) The site‐specific extended haplotype homozygosity (EHH) statistic for region 1 (F) and region 2 (G), with blue lines corresponding to resistant haplotypes and red lines denoting susceptible haplotypes. The black dotted line indicates the focal SNP included in each analysis (region 1: 99,802,212; region 2: 160,178,236).

By scanning the genome for SNPs with elevated *F*_ST_, we detected two regions that were highly differentiated between resistant and susceptible individuals (Figure S7). Importantly, both of these regions occurred on chr5, and they aligned with the two regions on that chromosome identified from the GWAS (Figure 4). Hereafter, we define these two regions as region 1 and region 2, based on their position on chr5 (region 1: 99.7 Mb, and region 2: 160.1 Mb). Although neither region contained differentially fixed sites (i.e., *F*_ST_ = 1), *F*_ST_ did exceed 0.7 at three sites in region 2, indicating extensive sequence divergence between resistant and susceptible populations.

Further inspection of these two regions revealed that the divergence between susceptible and resistant populations was caused primarily by a reduction in diversity and polymorphism in resistant, but not susceptible populations (Figure 4D,E). Haplotype diversity was 45% lower in resistant individuals than in susceptible ones in region 2 (resistant: 5.4 × 10^−4^ ± 2.7 × 10^−5^ S.E.; susceptible: 7.9 × 10^−4^ ± 2.9 × 10^−5^ S.E.). Although diversity was similar between susceptible and resistant populations in region 1 (resistant: 2.8 × 10^−4^ ± 1.9 × 10^−5^ S.E.; susceptible: 2.9 × 10^−5^ ± 1.6 × 10^−5^ S.E.), the proportion of segregating sites among resistant haplotypes was nearly half that of susceptible individuals in both regions (region 1: resistant = 0.54, susceptible = 0.93; region 2: resistant = 0.61, susceptible = 0.93). These patterns were in stark contrast to the proportion of segregating sites across all chr5, where polymorphism was much more similar between resistant and susceptible groups (resistant = 0.77, susceptible = 0.85). Finally, resistant individuals showed a frequency of the alternate (i.e., non‐reference) allele that was nearly twice as high as that of susceptible individuals in both regions (average frequency among sites for region 1: resistant = 0.22, susceptible = 0.11; region 2: resistant = 0.19, susceptible = 0.10). This difference is even more pronounced when we consider the number of variants where the alternate allele has a frequency greater than 0.5. In region 1, there were 37 sites where the alternate allele was at a frequency > 0.5 in resistant individuals, but only 19 sites in susceptible individuals. In region 2, there were 100 sites in resistant individuals but only 8 among susceptible individuals. The presence of a high frequency of the alternate allele at multiple sites in these regions is consistent with selection increasing the frequency of distinct haplotypes in resistant but not susceptible individuals.

We also detected patterns of extended haplotype homozygosity in both regions that were more pronounced in resistant individuals. In region 1, we found a very broad region (roughly 300 kb) of relatively low EHH in resistant haplotypes, which can be contrasted with a very rapid decay in EHH among susceptible haplotypes (Figure 4). This implies positive selection and a selective sweep in resistant haplotypes that is old, was incomplete, or occurred from standing variation, as genetic diversity persists in resistant haplotypes. By contrast, the probability of homozygosity in region 2 was substantially elevated in resistant individuals relative to susceptible individuals, but this region extended only over 5 kb. This pattern implies recent and strong positive selection in resistant haplotypes, but the signal extends over a shorter interval, perhaps because of differences in local recombination rates. These two regions were also identified as having elevated H12 in resistant but not susceptible samples, confirming selective sweeps in both regions among individuals resistant to paraquat (Figure S8). In addition, windows in each region that were among the top 1% of H12 values contained some of the highest H2/H1 ratios, implying that multiple haplotypes appear to have increased in frequency in these regions. Thus, even though the patterns are different in each region, they are both consistent with selection driving an increase in the frequency of one or more haplotypes.

Finally, we also asked if the same regions of the genome were differentiated in all three resistant populations relative to the susceptible populations. To do this, we calculated *F*_ST_ in non‐overlapping 25 kb windows for all pairs of resistant and susceptible populations and counted in how many of these pairwise comparisons each window was found in the top 1% of the *F*_ST_ distribution. We found only two regions in the genome where all nine comparisons between resistant and susceptible populations showed the same window in the top 1% of the distribution, and these corresponded to regions within or directly adjacent to regions 1 and 2 (Figure S9). Indeed, there was a pileup of windows in these two regions where multiple comparisons showed elevated *F*_ST_. No other window across the genome exceeded six comparisons where that window was repeatedly in the top 1% of *F*_ST_ windows.

## 4 Discussion

Herbicide resistance is one of the greatest challenges in sustainable crop production, and elucidating the genetic basis of resistance could aid in improving weed management practices. *L. multiflorum* is one of the most troublesome weed species in the world, having evolved herbicide resistance in multiple cropping systems and countries. Herbicide resistance in *L. multiflorum* provides an invaluable opportunity to fill many fundamental knowledge gaps on the underlying genetic and ecological mechanisms of rapid, repeated adaptation to similar environments across empirical systems. A deeper understanding of the evolutionary dynamics of herbicide resistance may provide the framework to predict the development of resistance in weeds and inform management practices that can delay future resistance evolution.

The genomic resources that we generated in this work will be a valuable tool for dissecting the genetic bases of herbicide resistance and other traits. Obtaining a contiguous genome anchored to chromosomes is a crucial component of the assembly effort (Lee et al. 2016) that allows researchers to better understand the genomic context in which genes or variants of interest occur. The *L. multiflorum* genome assembled here had 90% of the DNA content placed on 7 chromosomes (*N*_50_ = 363 Mb), with 93% of the gene content complete, which is comparable to other high‐quality assemblies (Cai et al. 2023; Benson et al. 2023).

In this work, we identified large‐effect loci that were associated with the rapid evolution of paraquat resistance. It is often the case that phenotypic variation is controlled by many small‐effect loci (Pritchard and Di Rienzo 2010). However, under strong selection pressure, such as with herbicide use, rapid adaptation may require fewer large‐effect loci to shift populations to their new optima (Gomulkiewicz et al. 2010; Kopp and Matuszewski 2014). Such rapid shifts in phenotypic optima have been identified in organisms exposed to lethal chemicals, such as fish (Miller et al. 2024), insects (Ffench‐Constant 2013) and weeds (Giacomini et al. 2020). In the current study, we identified the genetic basis of paraquat resistance using multiple ‘‐omics’ datasets. Implementing multiple approaches has proven necessary to dissect the genetic architecture of plant traits (Du et al. 2018; Jiang et al. 2019), as any one experiment may have shortcomings (Li and Xu 2021). Overall, our QTL‐seq, GWAS, and population genomic approaches converged on two regions on chr5 that appeared to be associated with paraquat resistance in *L. multiflorum* . Furthermore, these are the only two regions in the genome where all nine pairwise *F*_ST_ comparisons between resistant and susceptible populations showed the same window in the top 1% of the distribution (Figure S9). These results imply that there is a parallel basis for this genetic architecture among the three resistant populations we sampled between Oregon and California. In addition, we identified significant SNPs on chr2 in the GWAS. Although this region might also contribute to resistance, we did not find any signal on chr2 for elevated *F*_ST_, suggesting that these associations could represent false positives (Platt et al. 2010; Kaler and Purcell 2019). Additional investigation into this region is warranted.

In other species, including in the weed *L. rigidum* that is closely related to *L. multiflorum* , paraquat resistance is believed to be caused by a single gene with incomplete dominance (Yu et al. 2009). By contrast, Shaaltiel et al. (1988) suggested the possibility of linkage between two genes involved in paraquat resistance. We found two linked loci on chr 5 that appear to be associated with resistance. One explanation for this finding is that there are genes in regions 1 and 2 that work in concert to generate the resistance phenotype. The resistant populations used in this study were collected from fields that have been subjected to recurrent paraquat applications, resulting in a constant selective pressure, suggesting that both loci may be necessary to achieve resistance to field doses of paraquat. However, recombination between these two regions would break up co‐adapted genotypes. Future experiments with larger sample sizes should explore patterns of linkage disequilibrium between these regions to investigate the possibility of epistatic selection maintaining particular genotypic combinations, as performed elsewhere (Kreiner et al. 2022; Gupta et al. 2023).

The population genomic analyses revealed molecular signatures that were consistent with the action of recent positive selection in resistant individuals. However, the patterns observed were different in each region. Specifically, region 1 showed no reduction in diversity in resistant haplotypes, and although there was a very broad pattern of extended haplotype homozygosity compared to susceptible haplotypes, the signal was low. Moreover, H2/H1 and H2 are both elevated in region 1. Together, these results suggest that there was positive selection in region 1, but it was either older, less intense, or occurred via selection on multiple haplotypes, resulting in the maintenance of genetic diversity. Given that paraquat has only been applied to agricultural fields for the past few decades, an older sweep does not appear likely, unless there was a pleiotropic effect of this region on a second trait that is no longer under selection. By contrast, region 2 shows a strong reduction in diversity in resistant haplotypes, as well as a strong signal of EHH relative to susceptible haplotypes, but this signal does not extend over a long genomic interval. This suggests strong positive selection in region 2, but it is possible that recombination has broken down the signal, or that the signal is heterogeneous among resistant populations. Indeed, we also detected an elevated H2/H1 ratio for windows in region 2, which could imply a soft sweep, perhaps with fewer haplotypes involved than in region 1. While future work along chr5 will be needed to address these hypotheses, we observed a pileup of windows in these two regions where multiple comparisons between resistant and susceptible populations showed elevated *F*_ST_ (Figure S9). When combined with the GWAS and QTL analyses, these data suggest that although the genomic consequences of selection in each region may differ, the set of populations we tested appeared to experience parallel effects of recent positive selection that led to the evolution of resistance.

Analysis of gene expression patterns produced valuable insights into how these plants responded to oxidative stress caused by paraquat. When all time points were analysed together, we observed an overall higher level of expression of *GSH2* in resistant plants from both F_3_ populations. Closer inspection at each time point provided more information into the temporal dynamics of this gene's expression (Table S4). Notably, we observed that *GSH2* was constitutively expressed at higher levels in the PRHC population (5‐fold) prior to herbicide treatment, but this same pattern was not found in the L60 F_3_ population. However, upon paraquat treatment, *GSH2* expression in resistant individuals increased to 17‐fold greater than the susceptible individuals in the L60‐derived F_3_ population. Although we observed two differentially expressed genes in common in both F_3_ populations, we cannot rule out the possibility of different resistance mechanisms among populations or additional small‐effect genes contributing to the phenotype, because each population also contained other differentially expressed genes associated with paraquat resistance. These results are consistent with the different evolutionary histories of L60 and PRHC (Brunharo and Streisfeld 2022). In our previous research, we observed that L60 and PRHC had distinct evolutionary histories, suggesting that they may have evolved different mechanisms to protect themselves from paraquat. The sample size we used in the GWAS only provides statistical power to identify large‐effect loci, so while the two regions on chr5 both appear as strong candidates, it remains possible that additional small‐effect loci also contribute to the resistance phenotype.

In addition to revealing the genetic architecture and evolutionary responses to resistance, our extensive genomic analyses identified promising candidate genes. Specifically, genes that encode NPF5.10 and DTX10 are of particular interest, as both are contained within the GWAS peaks and are adjacent to region 2 from the *F*_ST_ analysis. *NPF5.10* is also the only gene in these regions that was differentially expressed between resistant and susceptible individuals. It is a member of the NRT1/PTR family, which are nitrate and oligopeptide transporters localised to cellular membranes. It has been suggested that an amino acid substitution in the NPF6.4, a member of the NRT1/PTR family, reduced norspermidine (a polyamine) cell uptake in Arabidopsis (Tong et al. 2016). Polyamines are natural compounds responsible for protein synthesis, ion channel modulation and a number of biological processes (Igarashi and Kashiwagi 2010), and paraquat is known to competitively inhibit polyamine transporters (Hart et al. 1992a, 1992b). Brunharo and Hanson (2017) observed that paraquat‐resistant *L. multiflorum* pretreated with putrescine (a polyamine) became susceptible upon paraquat application, which suggested that a membrane‐bound transporter could be involved in the resistance mechanism. Restricted paraquat movement has been reported in resistant weed populations (Brunharo and Hanson 2017; Soar et al. 2003; Yu et al. 2010), which has been attributed indirectly to enhanced vacuolar sequestration of the herbicide by tonoplast‐localised transporters (Brunharo and Hanson 2017). Enzymes in the Halliwell–Asada cycle are involved in ROS quenching, and their increased activity can provide paraquat resistance. For example, in *Conyza bonariensis* , a broadleaf weed of wide distribution worldwide, increased expression of some of these enzymes was observed in resistant individuals (Ye and Gressel 2000).

We found two candidate genes that are members of the MATE family (DTX10 and DTX21) that are found in prokaryotes and eukaryotes. These transporters are localised to the tonoplast (Zhang et al. 2017), Golgi apparatus (Seo et al. 2012), and plasma membrane (Rogers and Guerinot 2002). A total of 58 MATE proteins have been annotated in *Arabidopsis* (Hvorup et al. 2003) and 53 in rice (Tiwari et al. 2014), and they have been shown to transport cationic compounds across cellular and organellar membranes (Kusakizako et al. 2020). Members of the MATE family may have many physiological functions and have restricted substrate specificities (Takanashi et al. 2014). They may be involved in the transport of secondary metabolites, ions, hormones and xenobiotics (Nimmy et al. 2022). Most importantly, an amino acid substitution in DTX6 has previously been shown to confer paraquat resistance in an experimentally derived *Arabidopsis* accession (Lv et al. 2021a). DTX6 localises to the endomembrane trafficking system and is responsible for increased vacuolar sequestration and cellular efflux of paraquat (Lv et al. 2021a). Similarly, DTX21 seems to confer herbicide tolerance by detoxifying glutathione‐conjugated xenobiotics and transporting them from the cytoplasm to the vacuole (Ramel et al. 2007). There are examples in the literature that successfully utilised rice (Saika et al. 2014) and Arabidopsis (Figueiredo et al. 2022) to perform functional validation of candidate genes identified in herbicide‐resistant weed populations, and those approaches could be used to further dissect the candidate genes identified in this work.

In this work, we elucidated the genetic architecture of paraquat resistance in *L. multiflorum* and identified promising candidate genes for future functional validation studies. In addition, we revealed different patterns of genetic diversity in two linked regions that were both associated with the resistance phenotype, consistent with positive selection driving an increase in the frequency of one or more haplotypes.

Understanding the genetic basis of herbicide resistance is crucial to improving the sustainability of cropping systems. A key component of weed management is to limit the dispersal of herbicide‐resistant individuals at multiple levels of the production operation, such as in the field, warehouses, or shipping containers during international trade. This could be accomplished with the development of quick resistance identification assays using genetic markers (Brusa et al. 2021), and the development of lateral flow assays that target these genetic variants (Baerwald et al. 2020). The genomic resources generated in this work will be valuable for a wide range of plant scientists. *L. multiflorum* is not only an agricultural weed, but there are also domesticated varieties that are cultivated as a cover crop and for forage, and the assembled genome could be used by breeders for trait improvement.

## Author Contributions

C.A.B. conceived and designed the study, collected data, assembled and annotated the genome, performed QTL, GWAS, RNA‐seq, and drafted and revised the manuscript. A.W.S. performed *F*_ST_ scans and haplotype networks and revised the manuscript. L.K.B. performed the WGCNA and revised the manuscript. M.A.S. performed *F*_ST_ and haplotype networks and drafted and revised the manuscript.

## Conflicts of Interest

The authors declare no conflicts of interest.

## Supporting information


Data S1.

**Table S3.**Genomic regions identified in GWAS and list of annotated genes.

**Table S4.**List of genes differentially expressed in the RNA‐seq experiment.

**Table S5.**WGCNA results for L60‐derived population containing module membership and gene trait significance.

**Table S6.** WGCNA results for PRHC‐derived population containing module membership and gene trait significance.

## Data Availability

Raw sequencing data is available at the NCBI Sequence Read Archive under BioProject PRJNA1046158. The genome and annotation files are available at the Weed Genomics Consortium at https://weedpedia.weedgenomics.org/. The scripts used for this work are available at https://github.com/caiobrunharo/ryegrass_genome_GWAS and https://github.com/awshort/ryegrass/tree/main. Benefits from this research accrue from the sharing of our data and results on public databases as described above.
