# Supplementary material for: The Genome of Lolium multiflorum Reveals the Genetic Architecture of Paraquat Resistance
Source: Mol Ecol. 2025 Apr 26;34(10):e17775. doi: 10.1111/mec.17775 (PMC12051776; doi:10.1111/mec.17775)
Supplement: Supplementary file 1 — Data S1. [file MEC-34-e17775-s004.docx]

**Supplemental Information for:**

**The genome of *Lolium multiflorum* reveals the genetic architecture of paraquat resistance**

Caio A. Brunharo, Aidan W. Short, Lucas K. Bobadilla, Matthew A. Streisfeld

**Table of Contents:**

| **Figure S1** | Page 2 |
| --- | --- |
| **Figure S2** | Page 3 |
| **Figure S3** | Page 4 |
| **Figure S4** | Page 5 |
| **Figure S5** | Page 6 |
| **Figure S6** | Page 7 |
| **Figure S7** | Page 8 |
| **Figure S8** | Page 9 |
| **Figure S9** | Page 11 |
| **Table S1** | Page 12 |
| **Table S2** | Page 13 |

^
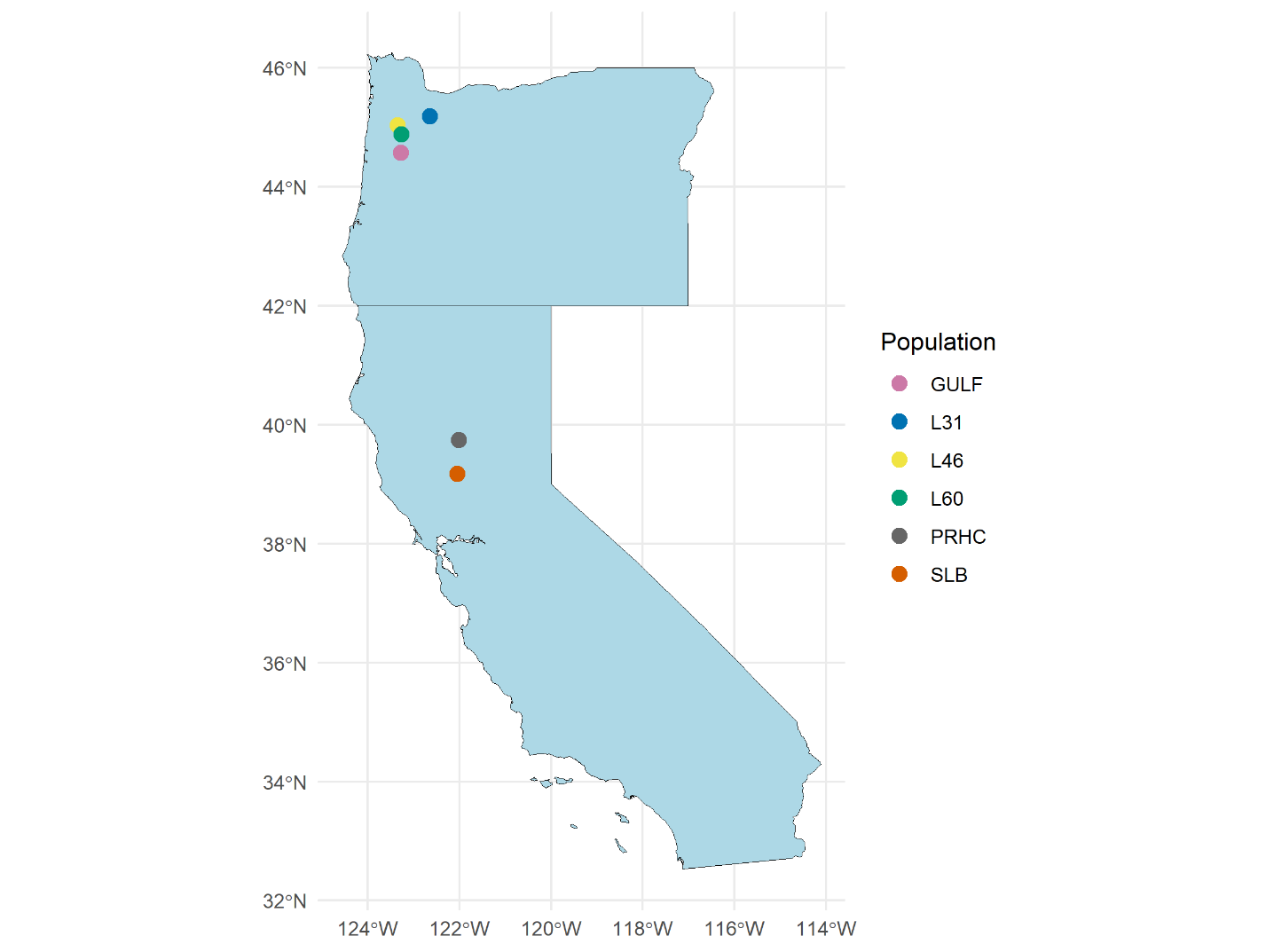
^

Figure S1. Map showing the origins of the populations used in our work. GULF, L46, and SLB are paraquat-susceptible, and L31, L60, and PRHC are paraquat-resistant.


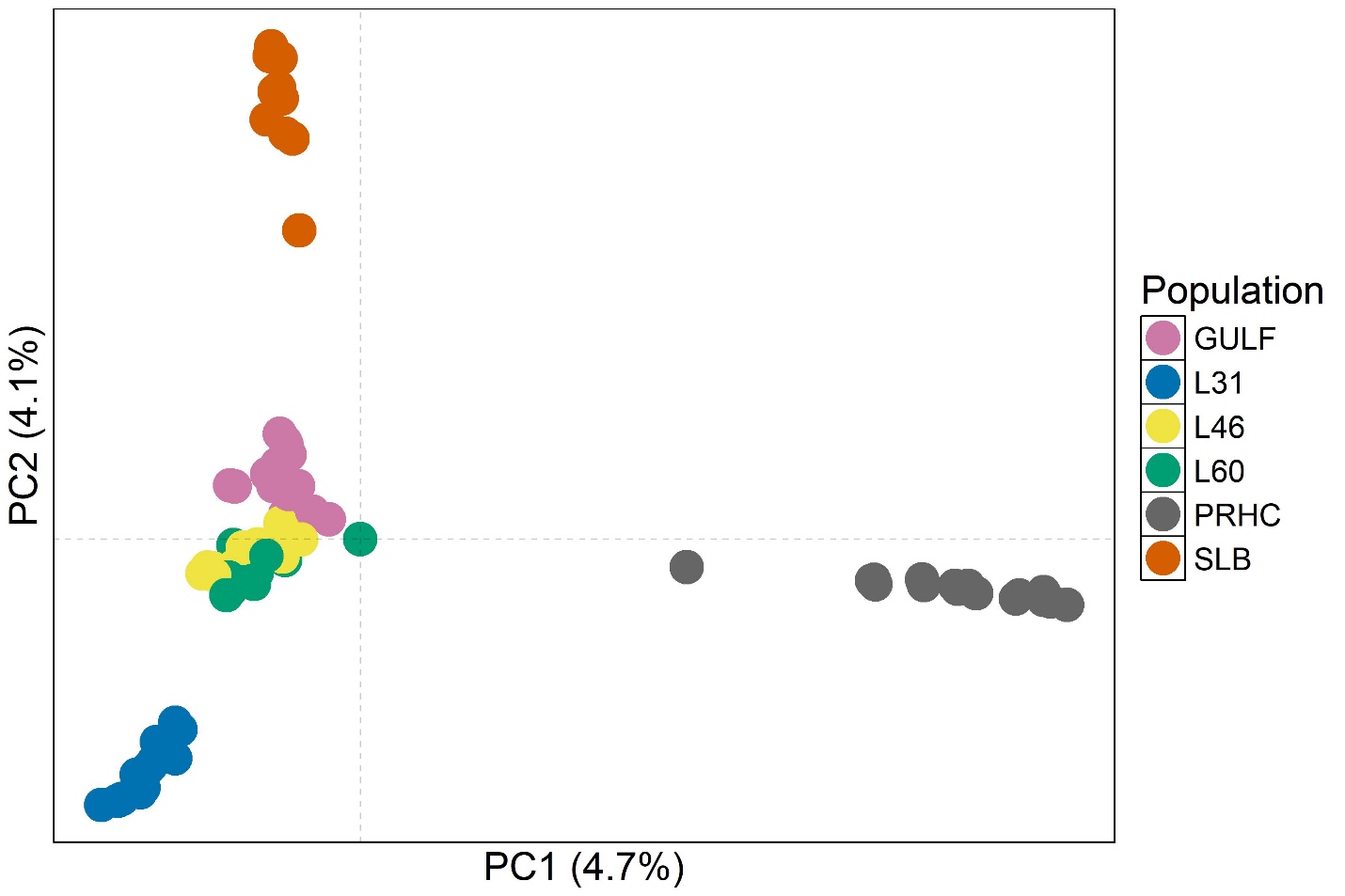


Figure S2. Principal component analysis of populations used in this study. GULF, L46, and SLB are paraquat-susceptible, and L31, L60, and PR are paraquat-resistant.

| 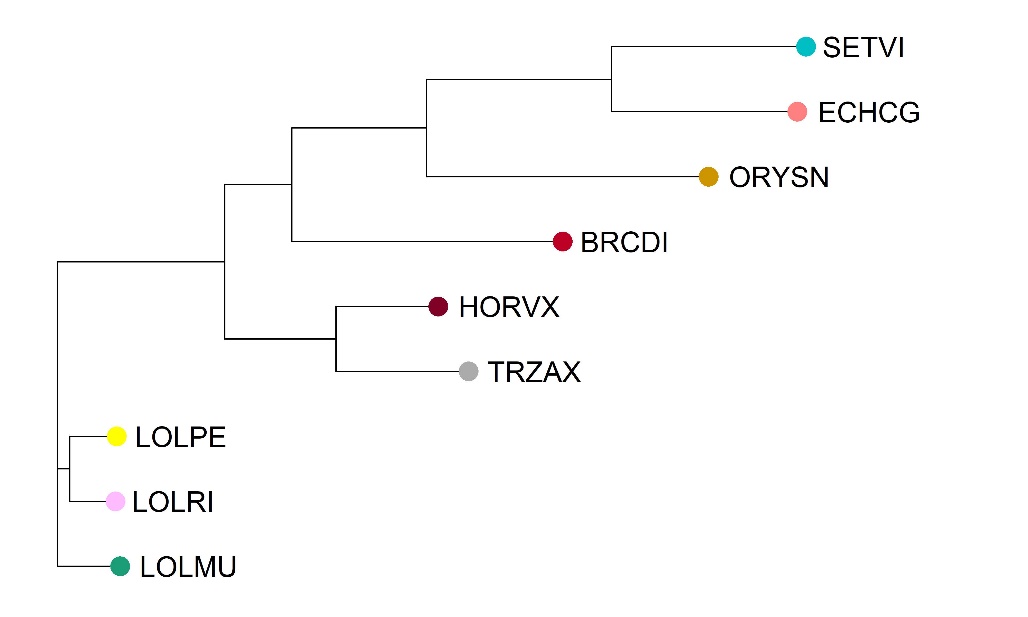  A  B |
| --- |
| 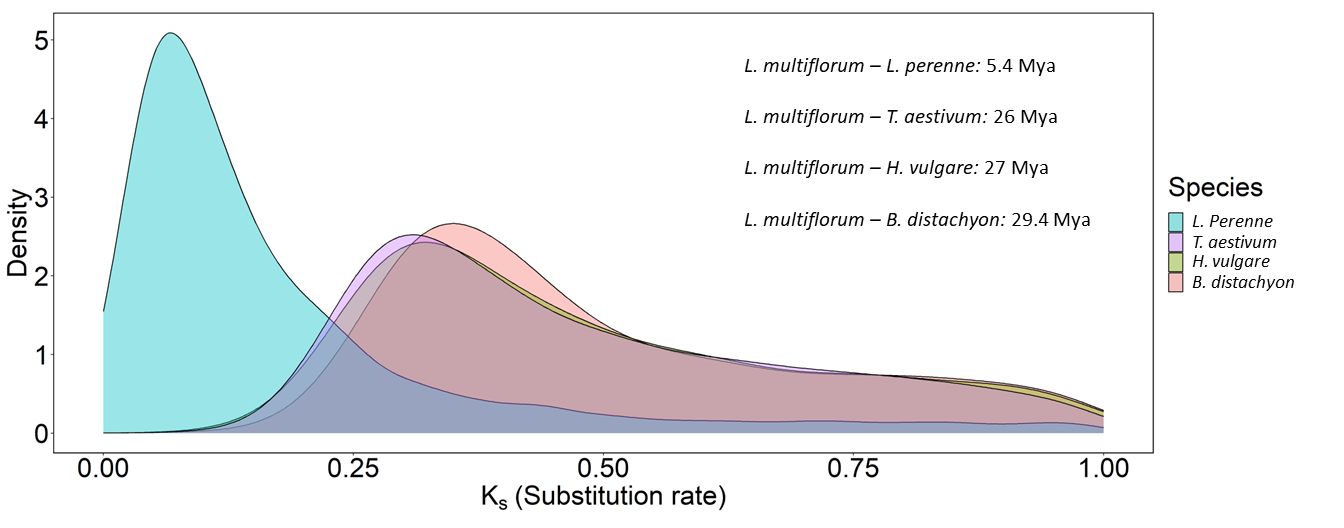 |

Fig. S3. Evolutionary relationships between *L. multiflorum* and other species in the Poaceae family. A) Phylogenetic tree of *L. multiflorum,* *L. perenne*, *L. rigidum*, *B. distachyon*, *T. aestivum*, *H. vulgare*, *S. viridis*, *E. crus-galli*, and *O. sativa*. B) Distribution of the synonymous substitution rate (K_s_) between *L. multiflorum* and closely related species.


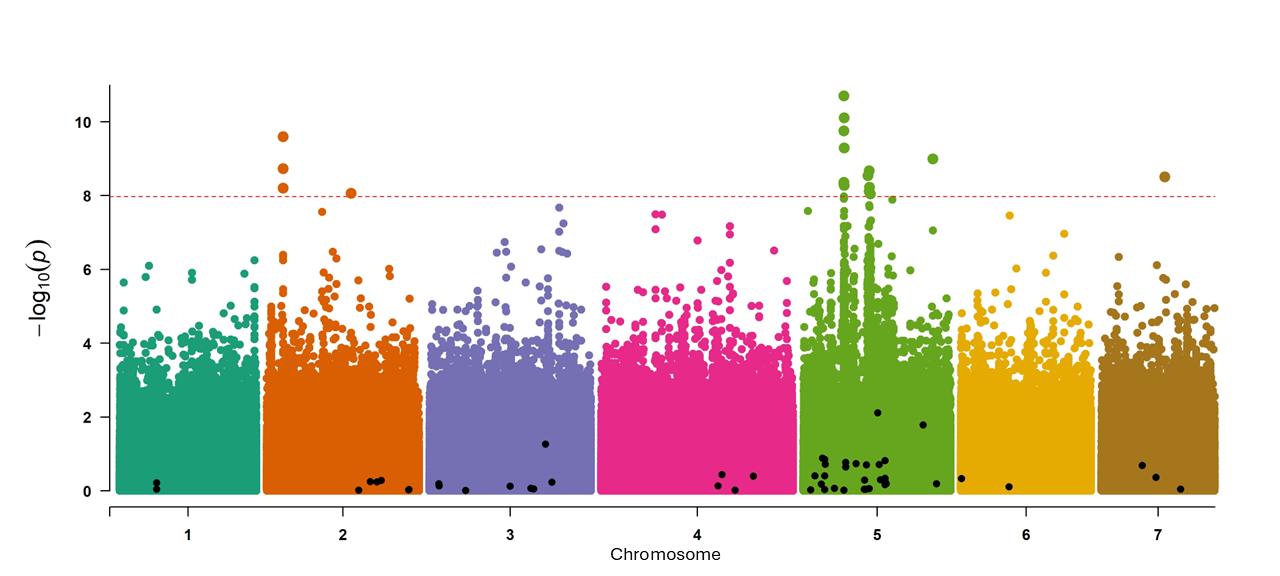
Fig. S4. Manhattan plot highlighting differentially expressed genes (DEG) identified in the RNA-seq study. Red circles are the SNPs closest to a DGE gene identified in the RNA-seq study.

| 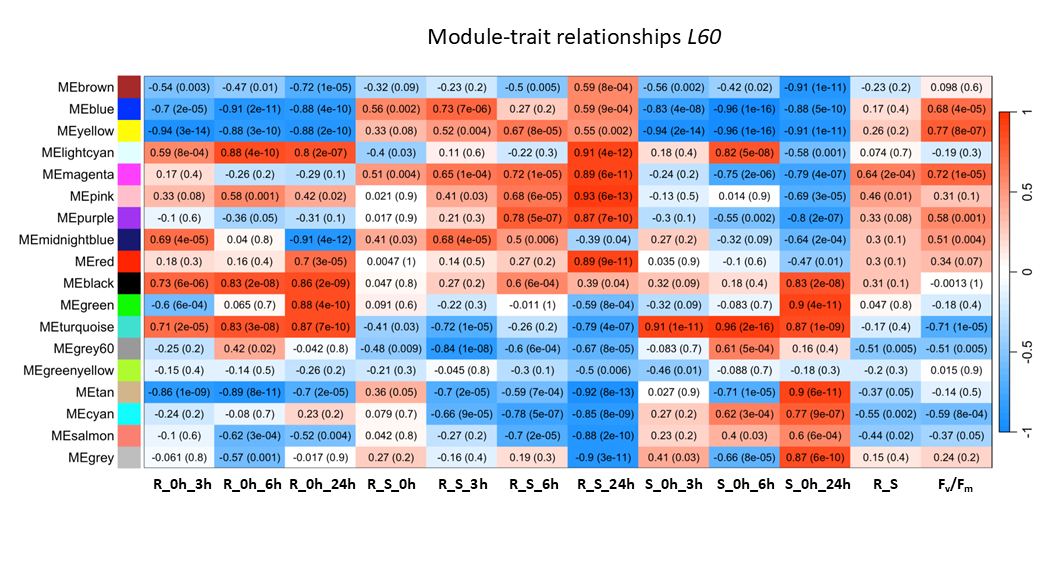  A |
| --- |
| 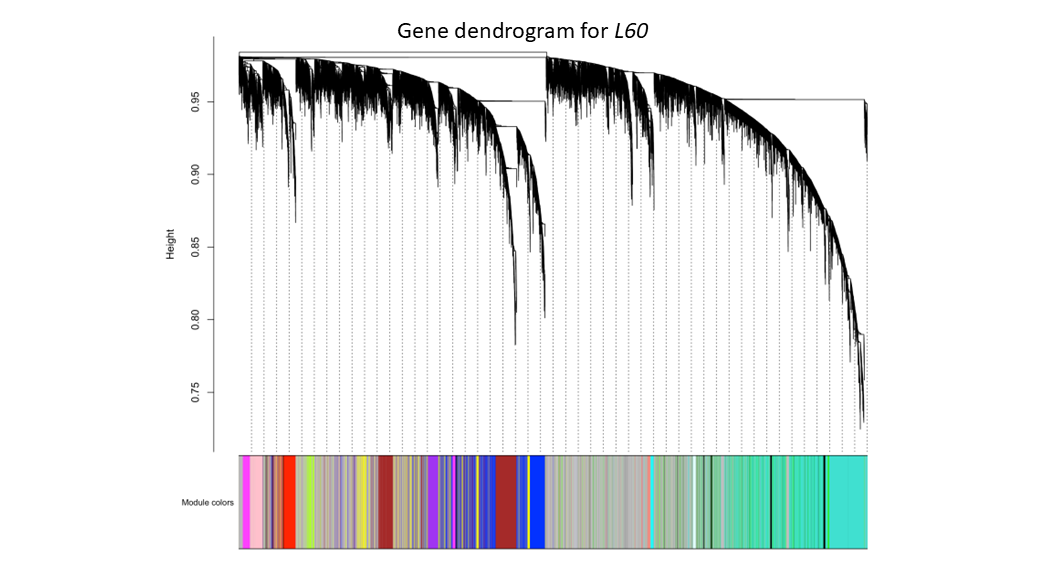  B |

Fig. S5. Weighted gene co-expression network analysis of the F_3_ paraquat-resistant L60 population. (A) Heat map of module-trait relationships shows a correlation from more negative (blue) to more positive (red) for each module, which are given names with different colors. Each column indicates a comparison between time points or individuals. (B) Hierarchical cluster trees show the co-expression modules identified by WGCNA.

| 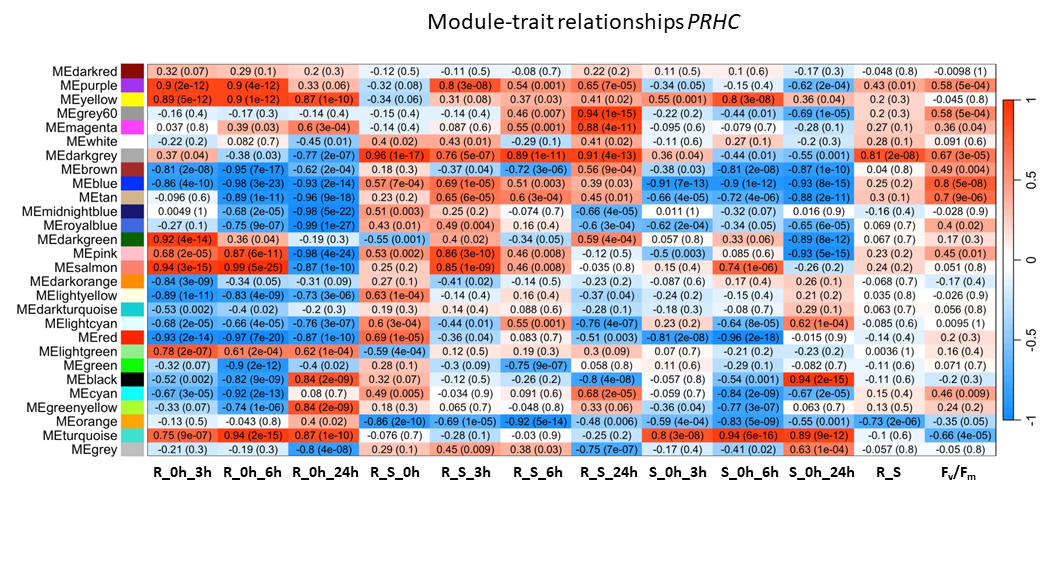  A |
| --- |
| 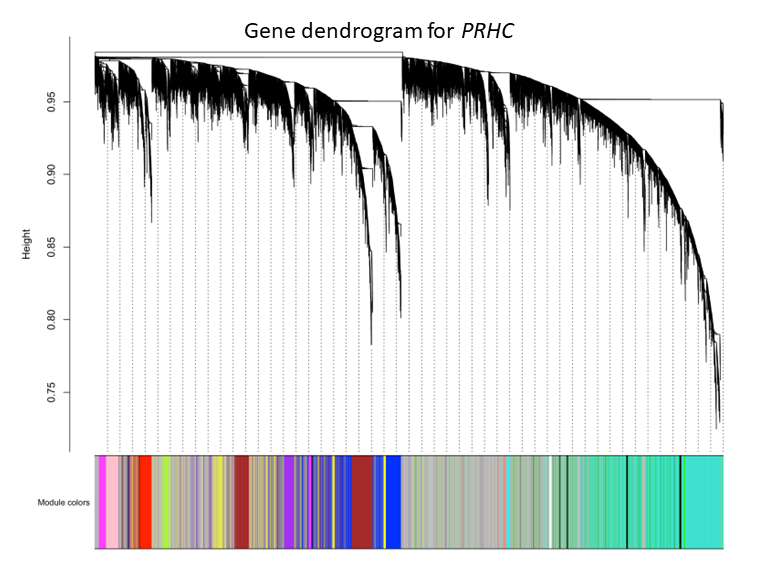  B |

Fig. S6. Weighted gene co-expression network analysis of the F_3_ paraquat-resistant PRHC population. (A) Heat map of module-trait relationships shows relationship from more negative (blue) to more positive (red) of each module color. Each column indicates a comparison between time points or individuals. (B) Hierarchical cluster trees show the co-expression modules identified by WGCNA.

Fig. S7. Genome scan of F_ST_ at all bi-allelic SNPs between paraquat-resistant and -susceptible populations of *L. multiflorum*.


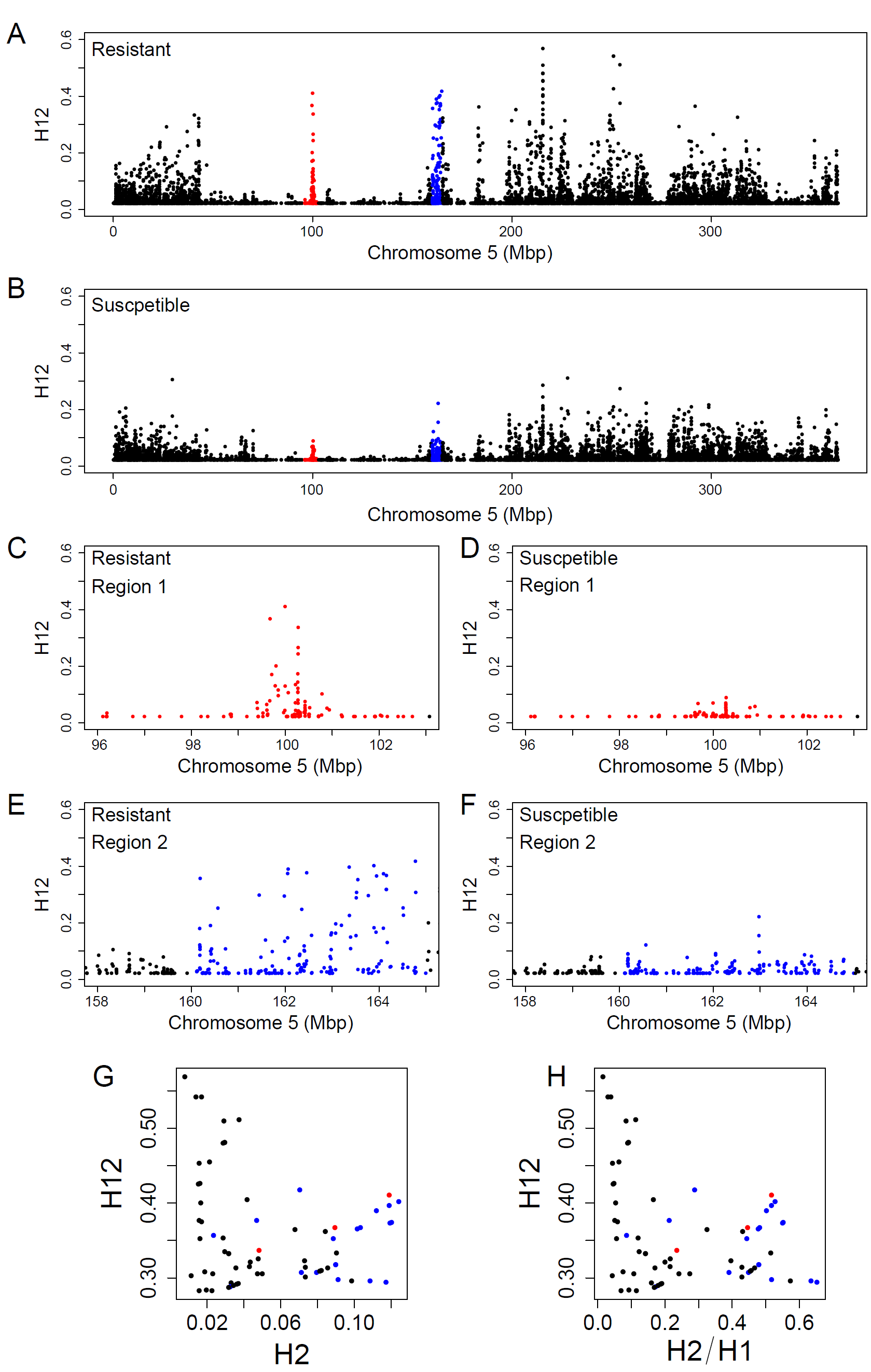


Fig. S8. Scans for selective sweeps along chromosome 5 using the H1, H2, and H12 tests. A) Genome scan of H12 along chromosome 5 from resistant individuals in 100 SNP windows, with 50 bp step sizes. Windows in region 1 are colored red and windows in region 2 are colored blue. B) The same genome scan as in panel A, but for susceptible individuals. C-F) Zoomed in plots of chromosome 5 for resistant individuals from region 1 (C), susceptible individuals in region 1 (D), resistant individuals in region 2 (E), and susceptible individuals in region 2 (F). G-H) Plot of H12 *vs* H2 (G) and H12 *vs* the H2/H1 ratio (H) for the top 1% of H12 windows along chromosome 5. Points are colored red if they are in region 1 and colored blue if they are in region 2.


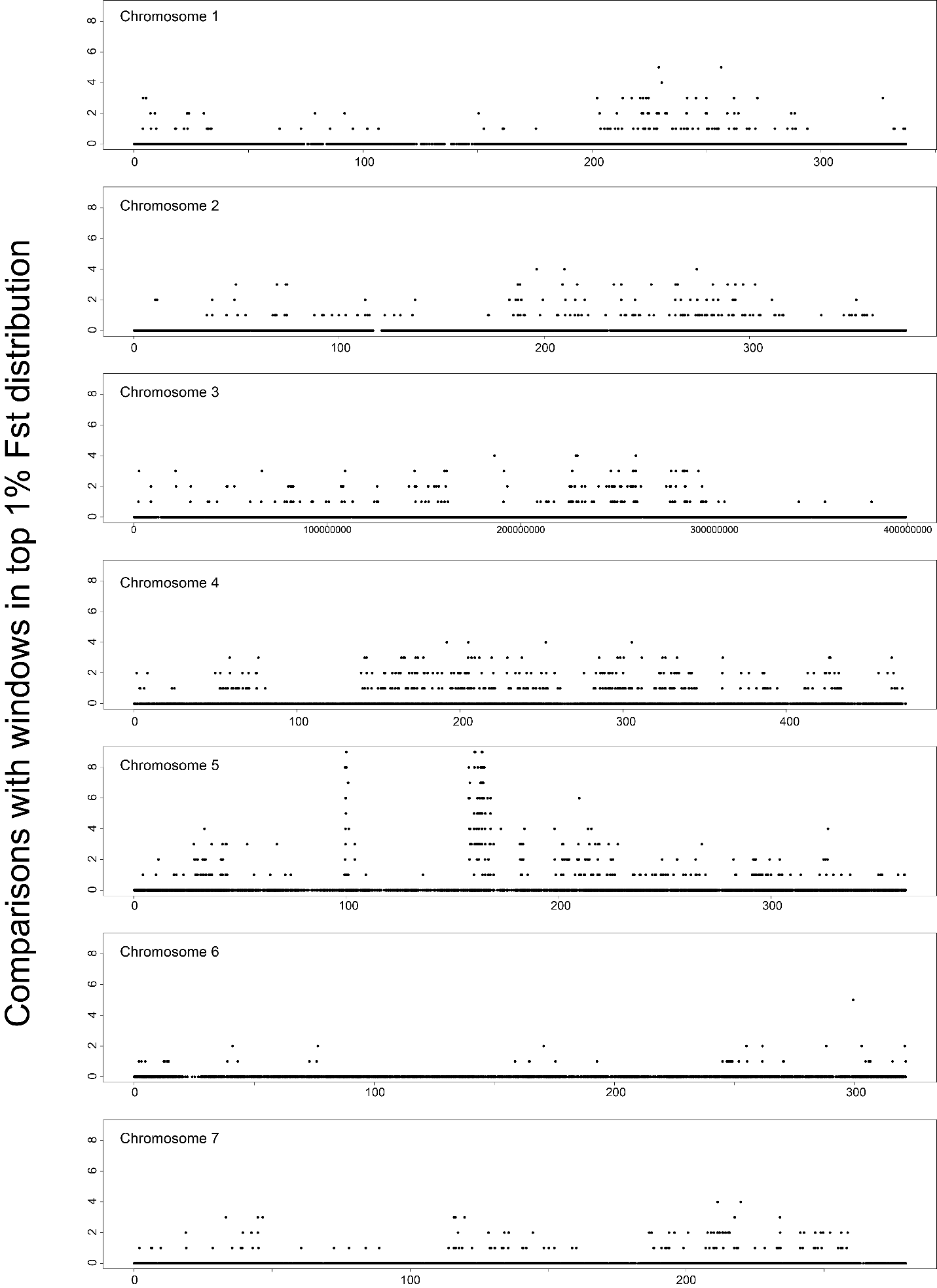


Fig. S9. The number of pairwise comparisons between resistant and susceptible populations where F_ST_ (calculated in 25 kb windows) was found in the top 1% of the distribution across windows.

Table S1. Summary of *L. multiflorum* assembly and annotation.

| **Characteristics** | **Value** |
| --- | --- |
| Genome size (bp) | 2,855,700,136 |
| Number of contigs | 253 |
| N_50_ (bp) | 363,560,625 |
| L_50_ (scaffold) | 4 |
| L_90_ | 7 |
| Repetitive elements (%) | 82.61 |
| Average gene length (bp) | 4,752 |
| Number of annotated genes | 49,295 |
| BUSCO | Single-copy: 72.5%  Duplicated: 20.3%  Fragmented: 0.2%  Missing: 7.0% |

Table S2. Repetitive element content in the *L. multiflorum* genome.

| **TE Class** |  | **Count** | **%** |
| --- | --- | --- | --- |
| LTR |  |  |  |
|  | Copia | 149860 | 4.80 |
|  | Gypsy | 1135594 | 35.04 |
|  | Unknown | 1449067 | 23.72 |
| TIR |  |  |  |
|  | CACTA | 328256 | 4.67 |
|  | Mutator | 180065 | 2.58 |
|  | PIF_Harbinger | 112106 | 1.85 |
|  | Tc1_Mariner | 127098 | 1.20 |
|  | hAT | 40264 | 0.51 |
| nonLTR |  |  |  |
|  | LINE_element | 10310 | 0.19 |
|  | unknown | 216 | 0.01 |
| nonTIR |  |  |  |
|  | helitron | 195580 | 3.18 |
| repeat_region |  | 138747508 | 4.86 |
|  |  |  |  |
| Total |  | 2358935386 | 82.61 |
